# Setting the Bases of the Photogenotoxicity of *p*‑Aminobenzoic Acid

**DOI:** 10.1021/acs.jcim.6c01438

**Published:** 2026-06-30

**Authors:** Julia Arnanz, Antonio Monari, Inés Corral, Juan J. Nogueira

**Affiliations:** † Departamento de Química, 16722 Universidad Autónoma de Madrid, 28049 Madrid, Spain; ‡ 555089 Université Paris Cité and CNRS, ITODYS, F-75006 Paris, France; § Institute for Advanced Research in Chemical Sciences (IAdChem), 16722 Universidad Autónoma de Madrid, 28049 Madrid, Spain

## Abstract

Despite its wide use in the past, the UVB filter *p*-aminobenzoic acid (PABA) is currently considered unsafe in the cosmetic industry. Among other reasons, there is the claimed formation of photoadducts with the nucleobases in DNA. We provide theoretical evidence showing the spontaneous intercalation of PABA in a (*dAdT*)_6_·(*dAdT*)_6_ strand. The π–π stacking interactions between PABA and the nucleobases result in an effective coupling of the native excited states of both molecules, altering the optical properties of DNA. Transition density matrix analysis shows that although the absorption spectrum of the DNA–PABA complex is dominated by excitons, the dark charge-separated states, which have been associated with photodamaging radical reactions in the literature, are overall more numerous in the entire absorption energy range. We show that PABA → DNA charge transfer states are more abundant, more energetically accessible, and potentially longer-lived than those across nucleobases, hence explaining the radicallic origin of the photoadducts between the filter and DNA.

## Graphical abstract

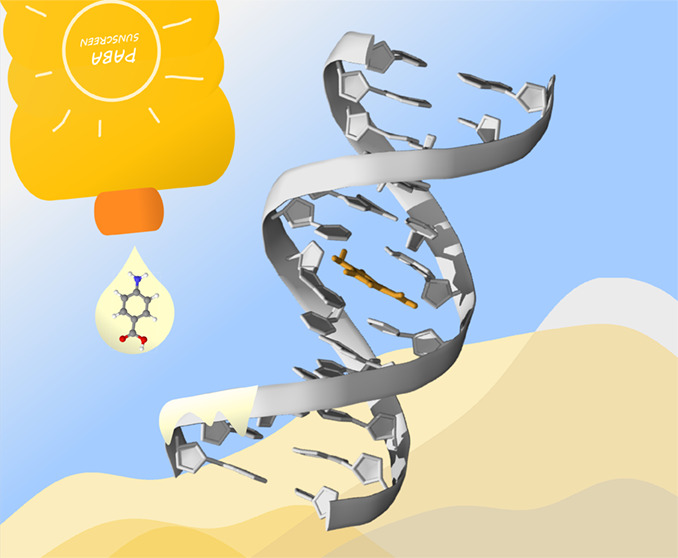

## Introduction

Ultraviolet (UV) radiation can trigger photoreactions in biomolecules and is mainly responsible for the acute and chronic effects of sunlight on the skin, such as erythema, photoageing, and photocarcinogenesis.^1,2^ In particular, the latter is explained by photoinduced mutations of the DNA sequence. UV light promotes reactions in the nucleobases, like the formation of cyclobutane pyrimidine dimers^3–5^ or 6–4 photoproducts.^6–9^ Additionally, endogenous or exogenous chromophores in tissues can promote photosensitized reactions that trigger the formation of reactive oxygen species that cause oxidative damage to the nucleobases.^1,10^ In this regard, sunscreen application is an interesting photoprotection strategy against such malignant outcomes of sun exposure. Organic molecules used as UV filters in sunscreens absorb the UV light, preventing it from reaching the tissues. These molecules must be photochemically and photophysically stable, that is, they should present nonradiative relaxation pathways that dissipate the absorbed UV energy thermally in a way that avoids the formation of unsafe photoproducts or the population of electronically excited states associated with photosensitized reactions.

*p*-Aminobenzoic acid (PABA), shown in Figure 1, is an efficient UVB light absorber (λ_max_ ≃ 286 nm);^11–13^ hence, it became one of the most used UV filters in sunscreen formulations during the second half of the 20th century.^14,15^ However, its protective abilities were questioned as dermatologists reported photoallergic reactions^16^ and in vitro studies showed photoreactivity with nucleobases (including PABA–thymine^17,18^ and photosensitized thymine–thymine adducts^19^ as well as radical products of the photodegradation of PABA^20,21^ ). These findings lead to a decline in its usage and, eventually, its consideration as a nonsafe substance for cosmetic products by the FDA and the European Union.^22,23^

**Figure 1:**
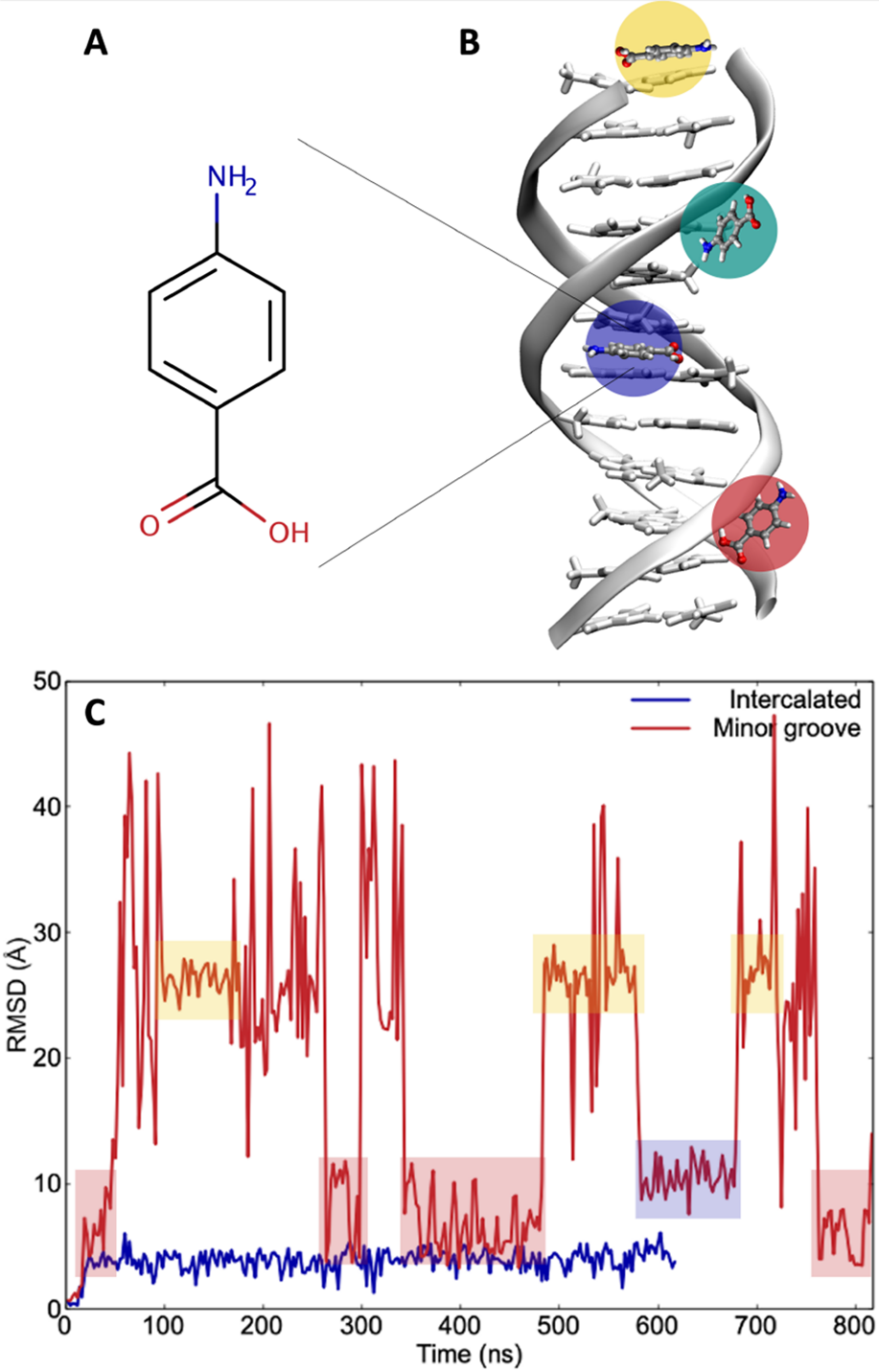
(A) Structural formula of *p*-aminobenzoic acid (PABA). (B) Representation of the (*dAdT*)_6_·(*dAdT*)_6_ strand showing the binding sites of PABA: intercalation (blue), major (green) and minor (red) groove bindings and stacking interactions with the terminal nucleobases (yellow). (C) RMSD of PABA during the trajectories starting from the intercalation and minor groove bindings. RMSD values are relative to each initial conformation. The sites explored during the MD simulation are highlighted in the same colors as in panel (B).

In spite of the above, to our knowledge, the photoreactivity of PABA and DNA has not yet been elucidated. In general terms, the formation of photoadducts can be traced back to charge or energy transfer processes.^18–20,24^ The charge separation between nucleobases and other chromophores following light absorption creates biradical species^25,26^ that can be extinguished via inter- or intramolecular reactions. Additionally, there may be triplet-mediated energy transfer reactions,^17,27^ whereby an accessible triplet state of the chromophore is quenched either by a nucleobase, thus sensitizing reactions therein,^18,21,27,28^ or by molecular oxygen, yielding the very reactive singlet oxygen, ^1^O_2_, which triggers the formation of radicals through reactive oxygen species.^28^

In this article, we aim to provide further insights into the first stages of the photogenotoxicity of PABA. In particular, we explored the binding of PABA to a model B-DNA double strand by means of molecular dynamics (MD) simulations, and we scrutinized the properties of the electronically excited states of the DNA–PABA complex by comparison to those of DNA alone. With this purpose, we employed static quantum mechanics (QM)/molecular mechanics (MM) techniques, where the QM part was treated at the time-dependent density functional theory level (TD-DFT). Resorting to transition density matrix analysis, we fully characterized the nature and distribution of the electronic excited states of the system, which is fundamental to understanding both the light absorption process and the subsequent deactivation dynamics.

## Computational Details

### Classical Molecular Dynamics

Considering that PABA can form adducts with thymine, we simulated a model DNA duplex alternating AT nucleotides. Since PABA can photosensitize the formation of cyclobutane adducts, we chose an alternating sequence to simplify a prospective study of the deactivation dynamics. The (*dAdT*)_6_·(*dAdT*)_6_ oligomer (i.e, an ATATATATATAT B-DNA double helix) was built using the nucleic acid builder (NAB) of the Amber20 software.^29^ PABA was placed manually on the major and minor grooves of the oligomer, while a script was coded to place PABA in the intercalated position, to ensure equal distancing to the four nucleobases and avoid repulsive instabilities. The *tleap* module contained in AmberTools20 ^29^ was used to surround the system with a periodic truncated octahedral water box, so that water molecules (described by the TIP3P solvation model^30^) are present within 12 Å from any solute atom. Twenty-two Na^+^ ions were added to neutralize the phosphate groups. The nucleic acids were described with the OL15 force field.^31^ The parameters in the second generation of the Generalized Amber Force Field (GAFF2)^32^ were used in modeling PABA, except for the restrained electrostatic potential charges, which were computed at the HF/6-31G* ^33,34^ level of theory on the geometry optimized with B3LYP/aug-cc-pVDZ^35^ in IEFPCM water,^36^ using the Gaussian 16 software package.^37^

Solvated DNA and solvated DNA–PABA MD simulations were run with this system setup. Short trajectories of 100 ns were run for the DNA duplex and the DNA–PABA system in the three binding modes using Amber20 software. Previously, the system was minimized using a steepest descent algorithm for 2500 steps and heated to 300 K during 30 ps. To prevent PABA from unbinding the DNA during the equilibration, its motion was restricted, introducing a harmonic potential of 10 kcal mol^–1^ Å^–2^. In these simulations, an NPT ensemble is generated, controlling temperature with the Langevin thermostat^38^ and pressure with the Berendsen barostat.^39^ Longer simulations were performed on the intercalated position (600 ns) and the minor groove (820 ns) using the NAMD software.^40^ These simulations were preceded by 10,000 minimization steps and an equilibration procedure where restrictions to the motion of PABA were gradually removed in 3 steps of 3 ns each, with decreasing force constant values of 1, 0.5, and 0.1 kcal mol^–1^ Å^–2^. Then, the NPT MD simulation was run at a temperature of 300 K, regulated by a Langevin thermostat,^38^ and pressure is maintained at 1 bar with a Langevin piston barostat.^41^

In all MD simulations, a time step of 2 fs was used, as the bonds involving hydrogen atoms were constrained by the SHAKE algorithm,^42^ and long-range electrostatics are treated within the Particle Mesh Ewald approach, considering nonbonded interactions up to 9 Å. The trajectories were processed and analyzed to obtain the time variation of variables like the root-mean-square displacement (RMSD) using the CPPTRAJ software.^43^

### TD-DFT/MM Absorption Spectra

The analysis of the optical properties of the DNA–PABA system was performed only for PABA intercalated between the nucleobases, as it is the most stable binding mode. To assess the effect of PABA on the excited states of the DNA–PABA complex, we first analyzed the absorption spectrum and density of states of the (*dAdT*)_6_·(*dAdT*)_6_ duplex alone. Subsequently, we inspected the modifications introduced by PABA. In these electrostatic embedding QM/MM vertical absorption spectra, the QM region comprises the 4 central nucleobases, two adenine–thymine pairs, and PABA, when present. The riboses, phosphates, and the rest of the nucleotides were modeled at the same MM level described above. Arguably, choosing a larger QM region would yield a more precise picture of the light absorption process, accounting for highly delocalized states. However, we estimated that this is an appropriate size to model the system in question, while maintaining a reasonable computational cost. This is also supported by experiments reporting that the effects of stacking on the excited states of DNA are already representative in stacked dimers, particularly for (*dAdT*)_*n*_·(*dAdT*)_*n*_ strands.^44,45^

Since the couplings between the nucleobases and, hence, the excited states formed upon light absorption strongly depend on the nucleobase arrangements adopted during the dynamics of the strand,^45^ we computed spectra of an ensemble of 100 equispaced snapshots from the 100 ns-MD simulations of the DNA and DNA–PABA systems, including the 30 lowest electronic excited states. The spectra result from the convolution of Gaussian functions centered on each excitation band, with heights proportional to the oscillator strengths (more details in Section S3). Given the size of the QM region and the objective of this research, that is, to contrast the spectroscopic properties of the DNA alone and with PABA bound, we consider it is justified to sample the configuration space using a statistically significant number of conformations obtained from classical trajectories instead of finer options such as Wigner samplings or QM/MM trajectories.^46,47^ In Section S3, we detail how we determined this statistical significance and show good experimental agreement of the spectrum of PABA in water solution, supporting this method (Figure S4).

The choice of DFT functional was based on the benchmark included in Section S2.1 of the Supporting Information, where we report the absorption spectra of PABA computed using several DFT functionals and their comparison with the experimental spectrum in water (Table S1). Although B3LYP^48^ performs slightly better in the description of PABA alone than the long-range corrected functionals sampled, CAM-B3LYP^49^/aug-cc-pVDZ^35^ was used in the QM/MM calculations, since long-range corrections are necessary to describe delocalized processes such as excitons or charge-transfer electronic states.^50^ Some studies claim that functionals with a higher long-range Hartree–Fock exchange contribution than CAM-B3LYP could lead to more accurate results in the description of local states;^51–54^ thus, to test the adequacy of this functional, we compared it to the second-order algebraic diagrammatic construction (ADC(2)) method by modeling the absorption of simplified PABA–thymine systems (see Section S2.2). We observe reasonable agreement of both theory levels, which validates CAM-B3LYP for these systems. Furthermore, this functional has ranked positively in benchmarks against ab initio excited state calculations of adenine–thymine systems,^52^ and has been used in similar studies on DNA.^26,55^

### Transition Density Matrix Analysis

We have characterized the four types of electronic excitations in DNA, which have been thoroughly studied in the literature:^24,26,54–57^ (1) Monomer-like excitations localized only in one nucleobase, (2) excitons, i.e., simultaneous local excitations in several nucleobases, (3) pure charge transfer (CT) excitations between nucleobases and (4) excimers, i.e., excited states with mixed locally excited and CT characters. A concise representation of these states is shown in Scheme 1A,B1. These definitions are extended to excitations involving PABA and are reflected in Scheme 1B2.

**Scheme 1:**
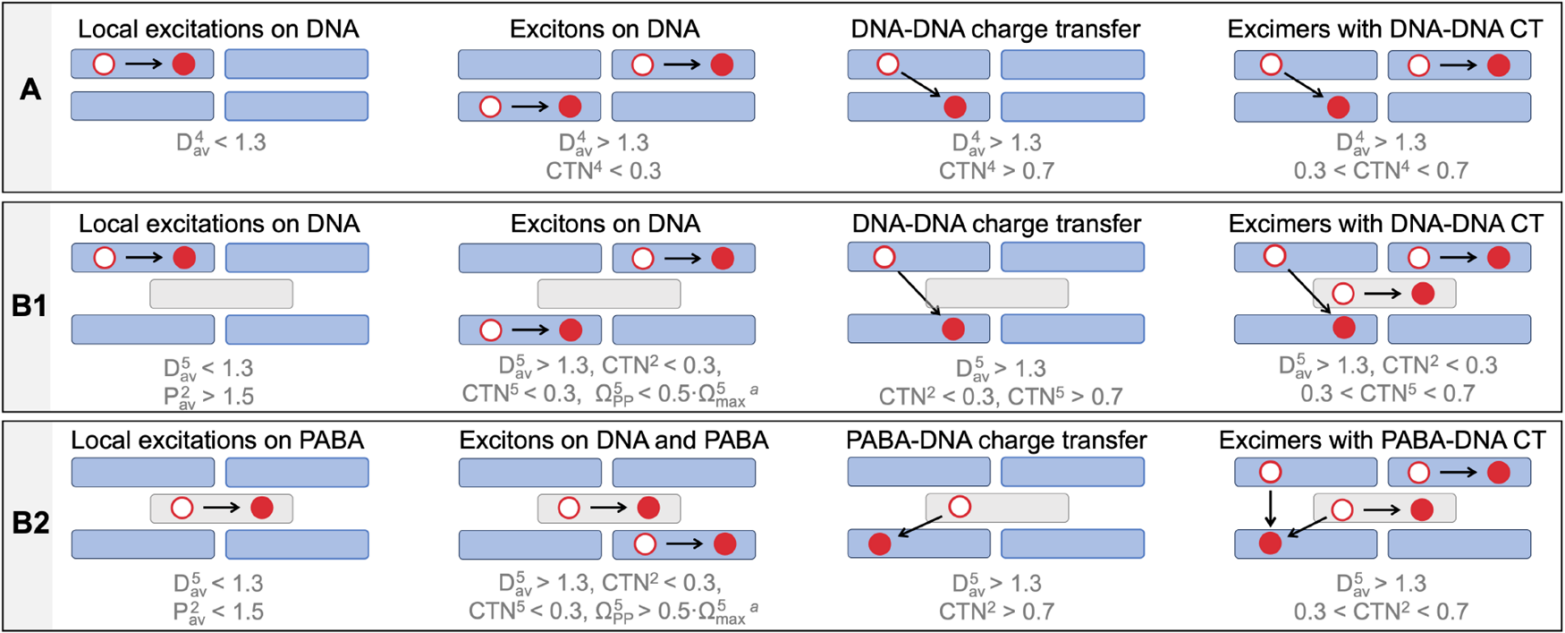
Classification of the Excited States of (A) Free DNA and the DNA–PABA Complex, Considering (B1) the States Involving Only DNA and (B2) States Involving PABA As Well^b^

a Ω_PP_^5^ is the coefficient of the local excitation on PABA (P) in the 5-fragment electron-hole correlation matrix of one excitation, and Ω_max_^5^ is the largest coefficient
b The criteria used for assigning each character are detailed below, where the superscripts indicate the number of fragments included in the analysis. The rectangles represent each of the components in the 4- or 5-fragment analyses and the colours, each of the fragments in the 2-fragment analysis (the bases in blue and PABA in grey).

The analysis and characterization of the excited states in the DNA spectrum were facilitated by the TheoDORE code.^58^ We established the criteria used to classify the transition density matrices upon visual inspection of the natural transition orbitals of several states, based on those used by Ibele et al.^26^ The wave function analyses of DNA and the DNA–PABA complex were performed by dividing the systems into, respectively, 4 and 5 fragments, one per nucleobase included in the QM calculation, plus PABA, in the latter. The average delocalization (*D*_av_) indicates the average number of fragments over which a transition is delocalized. Excitations were considered delocalized for *D*_av_ > 1.3 fragments. The charge transfer numbers (CTN) indicate whether a state is locally excited (CTN = 0) or completely charge-separated (CTN = 1) and are used to classify delocalized states in excitons (CTN < 0.3) and CT states (CTN > 0.7). Intermediate CTN (0.3 < CTN < 0.7) values correspond to excimers. Lastly, we identified the fragments involved in each excitation. For this, we used the positions of the hole and the electron in the case of charge-separated states, the average electron–hole position (*P*_av_) in the case of local states, and the coefficients of the electron–hole correlation matrices (Ω_AB_) in the case of excitons. Ω_AB_ coefficients measure the contribution of the electronic transition from fragment A to fragment B to the excited state. We refer to the Supporting Information for a detailed description of the former and the original TheoDORE paper^58^ for the mathematical grounds of the descriptors used in this work.

For the DNA–PABA system, we performed an additional wave function analysis, dividing the system into 2 fragments, one for PABA and another for the 4 nucleobases together. In this way, we used the CT contribution in the 2-fragment analysis (CTN^2^) to distinguish CT processes between nucleobases (CTN^2^ < 0.3) from CT processes involving PABA (CTN^2^ > 0.3). This is useful to discriminate the potential implication of PABA in photoinduced radical reactions. The criteria used in the wave function analysis of DNA and the DNA–PABA complex are summarized in Scheme 1.

## Results and Discussion

### PABA Binding to DNA

Organic ligands are known to establish noncovalent interactions with DNA in at least three ways: intercalated binding, whereby the ligand is stabilized by π–π stacking interactions with the nucleobase pairs, or the two groove bindings, where it establishes hydrogen bonds and electrostatic interactions with the polar groups in the major or minor grooves.^59,60^ These three are illustrated in blue, green, and red in Figure 1, respectively. Since the stability of these binding modes depends on the nature of the ligand, we assessed the binding strategy of PABA by placing it on each of these sites. It must be noted that the carboxylic group of PABA is expected to be predominantly deprotonated under physiological conditions, based on its aqueous p*K*_a_ value of 4.87.^61^ However, the negatively charged phosphate backbone of DNA makes binding of the anionic form less favorable, particularly in the groove regions (Figure S1). In the case of intercalation, the more hydrophobic environment between stacked base pairs may stabilize the neutral form to a greater extent than in aqueous solution. Therefore, a shift of the acid–base equilibrium toward the neutral species within the DNA binding area cannot be excluded. For this reason, we have focused here on the neutral form of PABA as a simplified model to investigate its possible interaction modes with DNA.

The major groove binding was shown to be unstable upon a short MD simulation (100 ns). Interactions therein are not sufficiently strong, and PABA alternates this weak binding site with water solvation. Longer MD simulations (600 ns for intercalation and 820 ns for minor groove binding) of the two remaining binding modes confirm the stability of the intercalated binding and the metastability of the minor groove binding. This can be seen in Figure 1C, which shows the average root-mean-square displacement (RMSD) value of PABA with respect to its initial position in each simulation. The weakly oscillating values of the RMSD during the trajectory of PABA intercalated between the nucleobases (blue line) indicate that PABA stays in that site throughout the entire simulation time, stabilized by π–π stacking interactions with the nucleobases. In contrast, the RMSD values of PABA in the minor groove binding simulation span over ∼50 Å because PABA explores different regions of the DNA strand. PABA remained in the minor groove for ∼250 ns in total (red area in Figure 1C), of which 140 ns were continuous (*t* = 340–480 ns), hence we consider this position as a metastable binding site. More interestingly, in the minor groove simulation, we see the spontaneous intercalation of PABA between the central nucleobases (blue area in Figure 1C). Although this observation requires more extensive sampling to properly characterize the intercalation process, it hints at a low energy barrier, which, together with the thermodynamic stability inferred from the intercalated simulation, reinforces intercalation as a preferential binding mode of PABA in the DNA. Furthermore, in the minor groove binding simulation, PABA also visited the water bulk and participated in π–π stacking interactions with the terminal nucleobases of the duplex (yellow area). These interactions are a simulation artifact, since in a longer DNA strand, terminal nucleobases would be less accessible; however, it could explain the binding to oligomers used for in vitro studies.^62^

### Optical Properties of DNA

The absorption spectrum of the model (*dAdT*)_6_·(*dAdT*)_6_ duplex shows a maximum at 5.42 eV. Transition density matrix analysis indicates that this band is mostly due to the population of excitons (Figure 2A). In fact, as illustrated in Figure 3A, excitons make up almost 50% of the states in the absorption spectrum, i.e., the distribution of states weighted by their oscillator strength, although they represent only 25% of the density of states (DOS), the unweighted distribution of states. To evaluate the extension of the delocalization of excitons, we used two different indicators: the average delocalization (*D*_av_, shown in Figure 4A) and the number of high-contributing fragments (in Table S2), that is, the number of fragments whose coefficients are at least half of the largest coefficient in the electron–hole correlation matrix. In excitons, this number is generally 1, meaning the excitation is localized on just one nucleobase with smaller contributions of other local excitations. In principle, these excitations resemble in character the local monomer-like excitations; however, the contribution to the excitation from the surrounding nucleobases, although small, suffices to alter the optical properties of the nucleobases with respect to the monomer-like equivalents (inset in Figure 2A). These 1-base-centered excimers represent 80% of the analyzed excitons (Table S2). In the remaining excitons, two (18%) and three (2%) nucleobases contribute equally to the excitation. This is supported by the distribution of the average delocalization, illustrated in Figure 4A, whose maximum *D*_av_ lies between 1.3 and 1.5. It must be noted that the distribution of the average delocalization is shifted to higher values compared to that of the high-contributing fragments because the contributions from low-contributing fragments shift the *D*_av_ distribution to higher values. This localization of the excitations on the monomers was observed in previous experimental^57,63^ and theoretical studies,^54^ and the authors attribute it to a poorer excitonic coupling in strands with alternating nucleobases, compared with homopolymers, where they observe larger delocalization extents.

**Figure 2:**
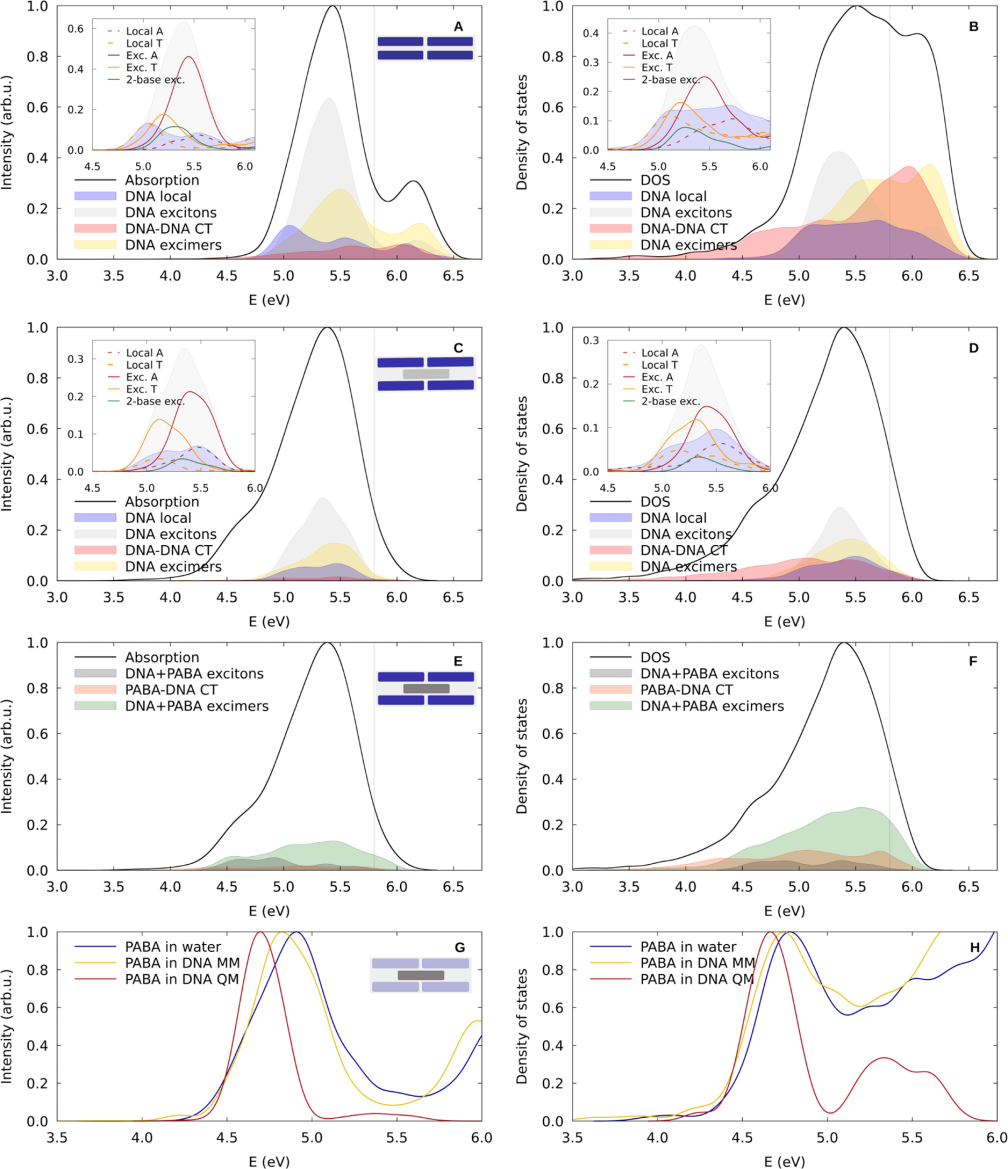
Decomposition of the absorption spectrum and density of states (DOS) of the (*dAdT*)_6_·(*dAdT*)_6_ duplex (A, B) and of the DNA–PABA complex (C–F) calculated from 100 vertical spectra at the QM­(CAM-B3LYP/aug-cc-pVDZ)/MM level of theory. QM regions are the 4 central nucleobases in (A) and (B) and the 4 central nucleobases and PABA in (C)–(F). The spectra of the DNA–PABA complex was divided to show the native states of DNA (C, D) and those with contribution from PABA (E, F). Absorption spectrum and DOS (G, H) of PABA in water solvation and in the DNA–PABA complex with and without the flanking nucleobases included in the QM region. Outlined in every absorption spectrum are the fragments involved according to Scheme 1. The insets in (A)–(D) show the contributions of each nucleobase to DNA monomer-like states (dotted lines) and excitons (solid lines) centered in adenine (Exc. A), thymine (Exc. T) or 2 bases (2-base exc.). The spectra of DNA and the DNA–PABA complex are comparable up to 5.80 eV (vertical gray line) (see text). Intensity (in arbitrary units) and DOS are scaled to 1.

**Figure 3:**
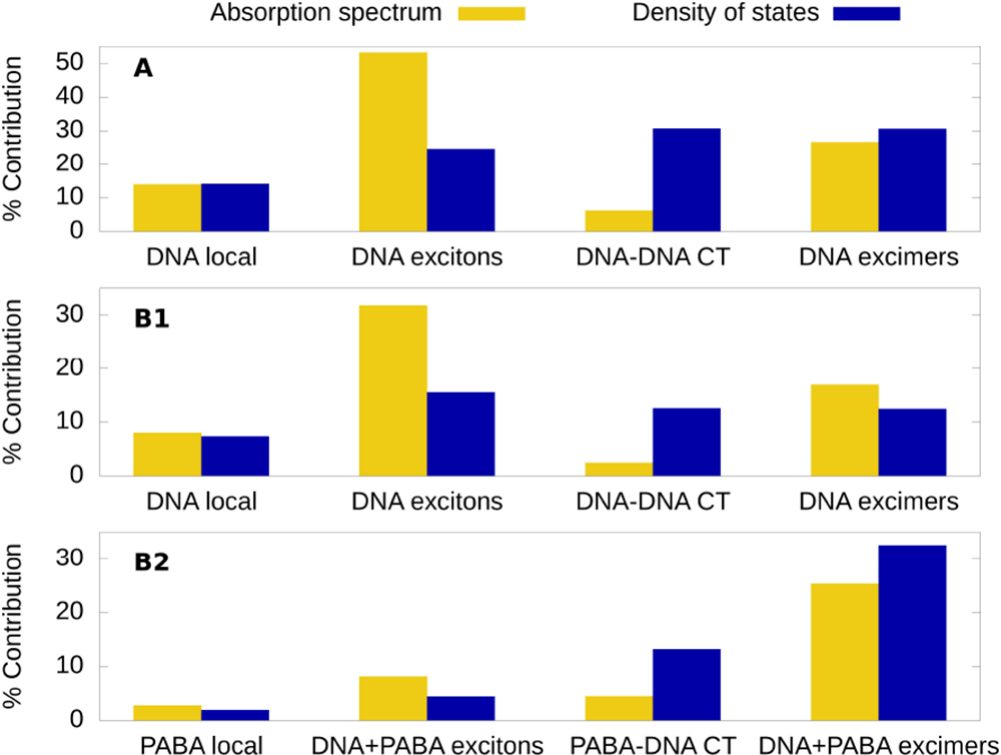
Relative contributions of each type of electronically excited state to the absorption spectra and DOSs of (A) the DNA strand, the DNA–PABA complex, considering (B1) the native states of DNA and (B2) states involving PABA. The excitations are those represented in Scheme 1.

**Figure 4:**
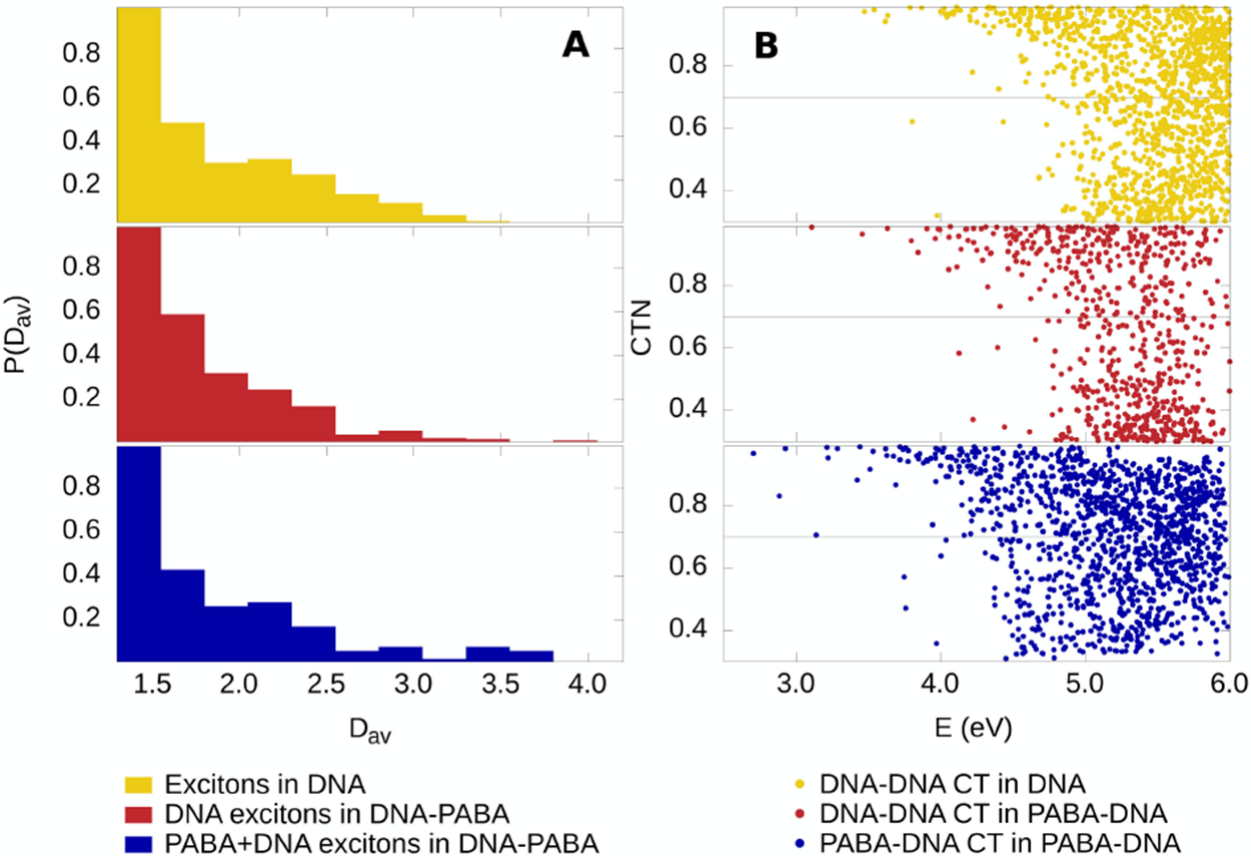
(A) Distribution of the average delocalization of excitons in DNA and the DNA–PABA complex (*D*_av_^5^). (B) Distribution of the CTN with respect to energy in pure CT states (above the horizontal line) and excimers (below).

In the (*dAdT*)_6_·(*dAdT*)_6_ strand, the delocalization of the excitation shifts the band of the adenine-centered excitons—those where the only high-contributing fragment is one adenine base—to lower energies compared to their monomer-like equivalents (from 5.54 to 5.45 eV, see solid and dotted lines in the inset in Figure 2A), while it shifts the thymine-centered exciton band to higher energies (from 5.05 to 5.20 eV, also in the inset in Figure 2A). In consequence, the band of all excitons is narrower compared to that of local states (gray vs blue shades in Figure 2A,B), as observed in the 5.00–5.70 eV range of both the spectra and the DOS. Interestingly, adenine and thymine do not contribute to the same extent to the population of the monomer-like excited states, with a greater contribution of thymine to the absorption spectrum and the DOS (red and orange dotted lines in the insets of Figure 2A,B). The converse is observed for excitons (solid lines in Figure 2A,B): excitations involving adenine are more abundant than those involving thymine, perhaps because the larger π system of adenine favors the delocalization of the excitation over more nucleobases via π–π stacking.^24,45,64^

Charge separation promotes DNA lesions mediated by radical reactions.^6,25^ Thus, analyzing the accessibility and stability of DNA CT states is key to predicting photodamage. Even though the DOS of pure CT states and excimers show identical abundances of both types of excited states (30.6 and 30.7%, respectively, as illustrated in Figure 3), we observe different absorption intensities, contributing each in 8 and 30% to the spectrum, as a result of the poor molecular orbital overlap of the fragments involved in pure CT states. The DOS of CT states peaks at 5.97 eV (Table 1). As energy decreases, their abundance is steadily reduced until 4.50 eV. A small fraction of CT states covers the 3.20–4.50 eV range of energies, with their number decreasing concomitant to the energy, as can be seen in Figure 2B. This tail in the absorption spectrum has been reported in experimental studies on DNA strands, where it was assigned to excimers rather than pure CT states, because of their perceptible absorption intensity.^25^ Excimers, on the other hand, combine spectral features of both CT states (high DOS at high energies) and excitons (local maxima in the DOS from the adenine-centered at 5.60 eV and from the thymine-centered at 5.11 eV). Whereas in pure CT states lower energy values are associated with higher CTN, energy is independent of the CTN in excimers, as illustrated in the distributions in Figure 4B. Moreover, analysis of the electron–hole correlation matrices (Figure S6) shows that in pure CT states, the dominant excitations are A → T, and the amounts of these CTs are comparable across stacked and paired bases. Diagonal A → A or T → T transfers (i.e., CTs to the base paired to the stacked nucleobase) are also relevant in the DOS. These findings diverge from those in the TD-DFT study from Lange and Herbert,^54^ who report no interstrand CT below 6.7 eV. However, this may be attributed to the lack of DNA conformational sampling in their absorption spectra, leaving out conformations where more effective couplings facilitate the charge transfer. The preference of A → T excitations over T → A is coherent with the largest vertical electron affinity of thymine (−1.55 vs −1.15 eV, as estimated for the nucleobases solvated in CPCM water at the UKS-CAM-B3LYP/aug-cc-pVDZ level)^65^ and the lower vertical ionization potential of adenine (6.31 vs 6.71 eV). The vertical ionization energies and electron affinities of all the molecules are provided in Table S3. These preferences also appear in excimers where A → T transitions are combined with local excitations, mostly on the adenines.

**Table 1:** Band Maxima of the Different Contributions to the Absorption Spectra and DOS^a^

	absorption spectrum		DOS	
	DNA	DNA + PABA	DNA	DNA + PABA
DNA local	5.05 (0.10)	5.47 (0.07)	5.69 (0.15)	5.50 (0.10)
PABA local		4.70 (0.04)		4.57 (0.04)
DNA excitons	5.40 (0.46)	5.35 (0.33)	5.35 (0.42)	5.36 (0.29)
DNA + PABA excitons		4.91 (0.05)		5.38 (0.04)
DNA–DNA CT	6.03 (0.04)	5.46 (0.01)	5.97 (0.37)	5.08 (0.09)
PABA–DNA CT		5.17 (0.02)		5.07 (0.09)
DNA excimers	5.50 (0.20)	5.44 (0.15)	6.15 (0.37)	5.45 (0.17)
DNA + PABA excimers		5.44 (0.13)		5.55 (0.28)

a Positions of the bands are given in eV, and intensity, in parentheses, is relative to the corresponding maximum intensities of the complete spectra.

### Optical Properties of the DNA–PABA Complex

As expected, the DNA–PABA spectrum presents locally excited states, excitons, and charge transfer processes only involving the nucleobases, similar to those in the spectrum of the (*dAdT*)_6_·(*dAdT*)_6_ duplex alone. The decomposition of the absorption spectrum and DOS involving these DNA states is shown in Figure 2C,D, respectively. Although PABA is not explicitly involved, the DNA states are modulated by the chromophore, allowing us to evaluate the indirect effect of PABA in the optical properties of DNA. As in DNA alone, the monomer-like states contribute weakly both to the absorption (8.0%) and the DOS (7.4%, Figure 3B1). The band corresponding to the locally excited states of adenine (shown in the inset in Figure 2C) is shifted to lower energies, and that of thymine to higher energies, both by 0.05 eV compared to the absorption spectrum of DNA alone. The dark locally excited states, which appear in the DOS spectra at ∼5.5 eV, have larger shifts (∼0.20 eV) to lower energies in both nucleobases. The lowering in energy of these states, from a mere Franck–Condon perspective, might imply they are more accessible along the deactivation pathway, potentially resulting in a more diverse photophysics that compromises the photostability of the duplex,^24,64^ for example, by easing access to intersystem crossing funnels to the triplet manifold from which photosensitized reactions can take place.

It is also relevant to note that the presence of PABA alters the relative ratios of the locally excited bands, lowering the participation of thymine with respect to that of adenine in monomer-like states and increasing it in excimers (see insets in Figure 2A–D). This suggests that PABA–thymine π–π stacking in the DNA–PABA complex contributes to delocalizing the local excitations of thymine. Conversely, in excitons between nucleobases, the positions of the absorption and DOS bands are practically identical with and without PABA (∼5.34 eV), so no clear effect of PABA is observed in this regard (gray bands in Figure 2). We do notice, however, fewer excitons among stacked nucleobases, as the intercalation of PABA distorts the chain, increasing the distance between base pairs (Table S2). Although the differences in the exciton band are small, this poorer coupling translates into a decrease in the average delocalization of DNA excitons in the DNA–PABA complex with respect to DNA alone (red and yellow distributions in Figure 4A).

The relative abundances of DNA CT states, that is, states with charge separation between nucleobases, are slightly lower in the DNA–PABA complex than in DNA alone (Figure 3), and this is especially noticeable at higher energies (red bands in Figure 2). The introduction of PABA gives rise to new electronic transitions, which increase the density of the excited states. This entails that the 30 excited states that comprise the spectrum of the DNA–PABA complex span an energy range shorter than that of free DNA. For this reason, we will restrict the discussion to a range in which both spectra can be compared (*E* < 5.8 eV, indicated in Figure 2 with a vertical line). In the decomposed DOS of excimers (yellow area in Figure 2D), the presence of PABA introduces a shoulder at 4.90 eV, which we ascribe to CT processes across nucleobases, simultaneous to a small contribution from local excitations on PABA. Moreover, we notice a small DOS at energies as low as 2.00–3.10 eV (outside the scale of Figure 2D), absent in the DNA spectra, with no charge delocalized over PABA. This could hint at an indirect effect of PABA on improving the accessibility of CT states by decreasing their energy.

In the absorption spectrum of the DNA–PABA complex, there are a few bands associated mainly with the absorption of PABA, with little or no contribution from the nucleobases (local excitations on PABA in Scheme 1). These localized transitions participate in up to 2.8 and 1.9% to the absorption spectrum and DOS, respectively (PABA local in Figure 3B2). The CAM-B3LYP/aug-cc-pVDZ spectra on top of 100 snapshots from a 100 ns MD simulation of PABA surrounded by water molecules shows a peak at 4.90 eV, which is shifted to 4.85 eV when it is intercalated in the DNA strand. For comparison sake, at this point, we consider the whole DNA strand treated at the MM level. These spectra and DOSs are shown in Figure 2G,H, respectively. Intercalated binding creates a more hydrophobic environment around PABA in contrast to water solvation. Considering the larger dipole moment of the bright excited state compared to the ground state (5.36 vs 4.04 D, according to a static CAM-B3LYP/aug-cc-pVDZ calculation in vacuum), we would expect a hypsochromic shift when PABA becomes intercalated. Moreover, the restriction of out-of-plane motions of PABA resulting from intercalation also increases the absorption energy (see Supporting Information, Section S3). However, explicit nucleobase–PABA interactions stabilize the low-lying excited states of PABA, outbalancing the expected blueshift, resulting in lower absorption energies upon intercalation compared to water. This DNA-induced redshift is even more significant when the flanking nucleobases are included in the QM partition, bringing the absorption of the locally excited states of PABA to 4.69 eV. The intensity of these local states of PABA is in the scale to that of the monomer-like states of the nucleobases, as seen in Table 1.

These local excitations of PABA are generally coupled to the native excited states of the DNA. In this last section, we analyze the *active* role of PABA in modifying the light absorption of DNA. The same categories of delocalized states considered for DNA can be applied to these emerging states, shown in Scheme 1B2: excitons with local excitations over both PABA and the nucleobases and charge separation processes across PABA and the nucleobases.

Excitons delocalized over both PABA and the DNA are one-third of those only delocalized over the nucleobases (Figure 3). They are responsible for an additional band, absent in the spectra of free DNA and PABA, which, together with the purely local states of PABA and excimers with PABA–DNA CT, produce a shoulder on the absorption spectrum of the complex. This new band that appears in the range 4.50–5.00 eV (gray shade in Figure 2E,F) arises mostly from PABA-centered excitons with smaller contributions from the nucleobases, as well as from excitons with two high-contributing fragments: PABA and one thymine base, both with comparable contributions to the excitation. Simultaneous excitations of PABA with adenine also occur, but they absorb at ∼5.40 eV, the same as the peak of native excitons; hence, they contribute to the height of the maximum of the absorption spectrum of the DNA–PABA complex but do not introduce any additional features. As can be seen in Figure 4A, PABA couples effectively with the nucleobases, allowing an average delocalization of the excitation comparable to that across nucleobases in (*dAdT*)_6_·(*dAdT*)_6_, even reaching a non-negligible delocalization over more than 3 fragments (*D*_av_ > 3). Excited state dynamics studies show that DNA excitons promptly decay to monomer-like states from which CT states can be further populated.^26,57,64^ Assuming a comparable evolution in the presence of PABA, the introduction of new bright exciton states may open additional pathways leading to photodamage.

Charge-separation processes between PABA and DNA are more abundant than those between nucleobases (Figure 3). In particular, there are three times more excimers with PABA–DNA CT (32.6%) than with DNA–DNA CT (12.4%), considering their DOS. Such an abundance of these highly mixed states, together with the high average delocalization of excitons, proves the existence of a significant coupling between the excitations in DNA and PABA, which can be expected to have an intertwined photophysical evolution. As the analysis of the absorption spectrum and DOS in Figure 3 shows, both DNA–DNA and PABA–DNA pure CT states are equally abundant, but the latter are brighter, especially at energies below 5 eV (Figure 2C,E). However, because of their low transition dipole moments, we expect minimal direct population of any of these states upon light absorption, but rather from internal conversion from brighter higher-lying states. Excited-state dynamics studies from previous experimental and theoretical works on various DNA models show increased population of charge-separated states along the deactivation dynamics.^24,26,57,64,66^ Considering that excimers with PABA–DNA CT are ∼6 times brighter than their pure counterparts (25.4 vs 4.5%, in Figure 3), and that in the excitation pure CT states appear at lower energies than excimers (Figure 4B), if this state ordering is preserved upon deactivation, we could expect the charge separation initiated in excimers to evolve to purer CT character as the excited state relaxes. Moreover, as PABA–DNA excimers are more abundant and brighter than native DNA excimers both in DNA and in the DNA–PABA complex, we expect PABA to favor the formation of charge-separated states in strands rich in adenine and thymine, although this must be confirmed by further excited-state dynamics simulations.

We notice that the PABA → DNA charge transfer direction dominates over DNA → PABA (Figure S6B2). In fact, the latter is only observed in excimers, not in pure CT states, and the electron donor is mostly adenine. Regarding CTs from PABA to the bases, PABA → T transitions are more abundant than PABA → A. This is coherent with PABA having the lowest vertical ionization energy and thymine the largest vertical electron affinity, in absolute value (Table S3), which, in consequence, yields the lowest CT energy (Table S4). Moreover, we can associate the energy released in the inverse process, the quenching of the radical ions, with the proneness of the system to stay in the ionised forms, and hence, with the lifetimes of these states, although neglecting the kinetic aspects of the process. Therefore, a lower quenching energy could be qualitatively related to a longer lifetime of the radical ion species. Since the energy released when recovering PABA and T from PABA^**•**+^ + T^**•**–^ (−3.15 eV, in Table S4) is lower than that of any other combination of radicals potentially arising from CT excitations, we estimate that the PABA^**•**+^ and T^**•**–^, formed in the numerous PABA → T transitions, could be longer lived than the other CTs, including those between nucleobases, present in free DNA. Although more research, including the mapping of the charge-transfer deactivation pathways and molecular dynamics simulations of the subsequent deactivation stages, is still needed, this direct PABA → T electron transfer, favored by the electronic coupling between PABA and thymine, may provide a plausible mechanistic explanation for the nondimer thymine photoproducts reported by Aliwell et al., which are not observed in the absence of PABA,^18^ as well as for the PABA–thymine adducts and oxidative thymine lesions compatible with the involvement of reactive oxygen species formed upon radical scavenging by molecular oxygen,^17^ or with radical photodegradation products of PABA.^20^

## Conclusions

Overall, the results presented herein can explain the initial stages of the photogenotoxicity of PABA. First, we demonstrated that PABA binds a (*dAdT*)_6_·(*dAdT*)_6_ strand either by intercalating the nucleobase pairs spontaneously or by intermolecular interactions in the minor groove. Moreover, decomposition of the absorption spectra using transition density matrix analysis leads us to conclude that the absorption of light of the intercalated adenine and thymine nucleobases is altered when PABA is present, either by shifting the native excited states of DNA to lower energies or by forming new excited states with joint contributions of both molecules. Particularly, we have shown that PABA facilitates the formation of presumably longer-lived intermolecular charge-separated states readily upon light absorption and possibly during the deactivation of the system. Considering that charge separation is directly linked to photoinitiated radical reactions, we have proposed a mechanism that would trigger the formation of the PABA–nucleobase adducts and other thymine photoproducts reported in different experimental studies.^17–21^

## Supplementary Material



## Data Availability

Relevant data in this article, including input files for molecular dynamics simulations, absorption spectra and transition density matrix analyses are available at the Zenodo repository at https://zenodo.org/records/17278837.

## Notes

Additional details on the classical modeling of DNA–PABA interactions, benchmarking of TD-DFT functionals, configuration sampling absorption spectra, and analysis of the electronically excited states (PDF)
