# Supplementary material for: Setting the Bases of the Photogenotoxicity of p‑Aminobenzoic Acid
Source: J Chem Inf Model. 2026 Jun 30;66(13):7611–21. doi: 10.1021/acs.jcim.6c01438 (PMC13370781; doi:10.1021/acs.jcim.6c01438)
Supplement: Supplementary file 1 [file ci6c01438_si_001.pdf]

Setting the Bases of the Photogenotoxicity of *p*-Aminobenzoic Acid

Julia Arnanz,[a] Antonio Monari,[b], Inés Corral[a,c]
and Juan J. Nogueira[a,c]

[a] *Departamento de Química, Universidad Autónoma de Madrid, 28049, Madrid, Spain;*
E-mail: juan.nogueira@uam.es
[b] *Université Paris Cité and CNRS, ITODYS, F-75006, Paris France.*
[c] *Institute for Advanced Research in Chemical Sciences (IAdChem), Universidad Autónoma de Madrid, 28049, Madrid, Spain.*

Supplementary Information

1. Classical modelling of DNA-PABA interactions
2. TD-DFT functional benchmarking
3. QM/MM configuration sampling absorption spectra
4. Analysis of the electronically excited states
5. CT assessment through electronic energies

1 Binding of PABA$^-$

Considering that the carboxylic moiety in PABA can be deprotonated in the biological medium, we computed MD simulations to study the DNA binding of both the neutral and the anionic species. As expected, the electrostatic repulsion between the anion and the negatively-charged DNA strand prevents the binding, as the RMSD values during the simulations suggest (Figure S1). For this reason, we only address neutral PABA in this communication.

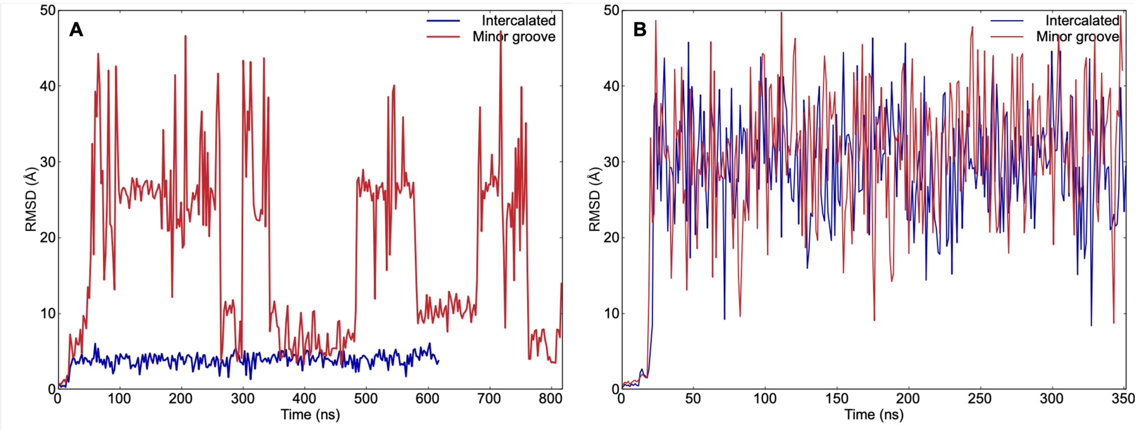

Figure S1: RMSD of (**A**) PABA neutral species and (**B**) PABA anionic species during the trajectories starting in the intercalated and minor groove bindings. RMSD values are relative to each initial conformation.

2 TD-DFT functional benchmarking

2.1 Comparison with experimental spectra of PABA

The choice of exchange-correlation functional and basis set was made upon comparison of several combinations to experimental spectroscopic data. B3LYP[1], CAM-B3LYP[2], M06-2X[3], ωB97-X[4] and ωB97-XD[5] functionals were benchmarked using cc-pVDZ, aug-cc-pVDZ and aug-cc-pVTZ[6] Dunning basis sets. We computed the absorption spectrum at each level of theory from the respective optimised structures. Both the optimisation and excited-state calculations were computed using CPCM-water solvation[7] in the Orca 5.0.1 software.[8]

Of the combinations analysed, shown in Table S1, the best agreement with experiment was obtained for the B3LYP functional. Nevertheless, considering our interest in charge-transfer states between PABA and the nucleobases, we selected the long-range corrected functional yielding the best agreement: CAM-B3LYP. We notice that the introduction of dispersion corrections does not impact greatly the accuracy of the results of the ωB97-X functional. Regarding the basis set, despite the noticeable differences when introducing diffuse functions (aug-cc-pVDZ vs cc-pVDZ), we see that the double-zeta set is accurate enough, compared to the triple-zeta. Thus, all the calculations shown in this Letter were performed at the CAM-B3LYP/aug-cc-pVDZ level of theory.

2.2 Comparison with ADC(2) spectra

In order to assess the adequacy of CAM-B3LYP to model the effect of the DNA-PABA interactions on the absorption spectrum of the complex, we compared it with the higher-

Table S1: Energies of the lowest-energy bright excited state os PABA for all functionals and basis sets considered. In parentheses are given the deviations, in eV, with respect to the experimental value, 4.38 eV

E (eV)	cc-pVDZ	aug-cc-pVDZ	aug-cc-pVTZ
B3LYP	4.78 (0.40)	4.51 (0.13)	4.55 (0.17)
M06-2X	5.16 (0.78)	4.87 (0.49)	4.92 (0.54)
CAM-B3LYP	5.11 (0.73)	4.81 (0.43)	4.86 (0.48)
ωB97-X	5.26 (0.88)	4.99 (0.61)	5.03 (0.65)
ωB97-XD	5.21 (0.83)	4.94 (0.56)	4.97 (0.59)

level second-order approximation to the algebraic diagrammatic construction (ADC(2)) method, as implemented in Orca 6.1.1.[9] Due to the large computational demand of these calculations, we simplified the system to PABA-Thymine dimers, with geometries taken from 6 equispaced snapshots of the intercalated PABA MD simulation. We chose thymine for this comparison to verify, especially, the correct description of the charge transfer (CT) states, estimated by CAM-B3LYP to be more significant for thymine than for adenine. In this 6 selected geometries we computed the ADC(2) and CAM-B3LYP vertical energies of the 15 lowest lying singlet states with the cc-pVDZ basis set, def2/J Coulomb-fitting auxiliary basis set[10] and the Coulomb-exchange and correlated auxiliary basis sets were generated automatically.[11] The resulting spectra —after combining the 6 spectra and convoluting Gaussian functions centred on each absorption band— is shown in Figure S2. We also included the decomposition in the main types of excitations in the PABA-Tymine dimer. We only performed the transition density matrix analysis for the CAM-B3LYP spectra. The character of the most relevant ADC(2) excited states were determined by inspecting the orbitals involved in the transition and using custom-made analysis programs.

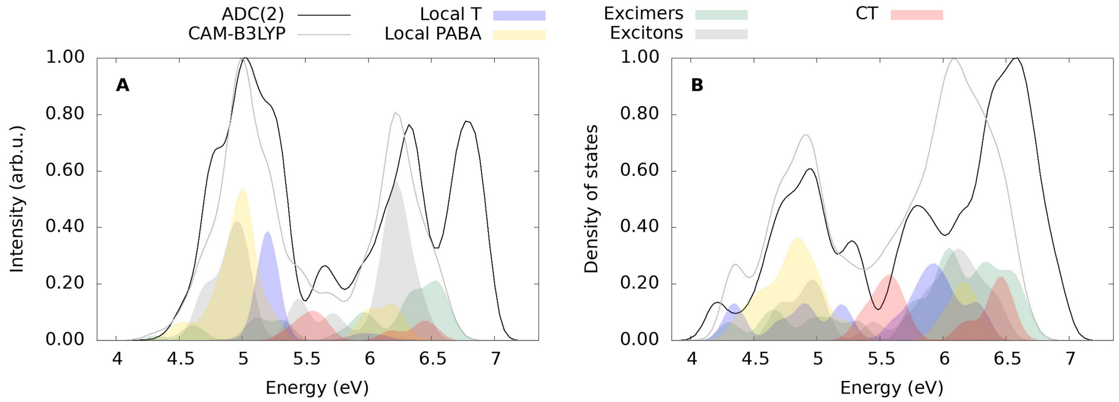

Figure S2: ADC(2) and CAM-B3LYP vertical spectra (A) and densities of states (B), and state-decomposition of the CAM-B3LYP spectrum and DOS. The CAM-B3LYP energies are redshifted by 0.3 eV to match better ADC(2). Absorption intensities and DOS are scaled to 1.

Overall, the shapes of both CAM-B3LYP and ADC(2) absorption spectra are comparable, with two distinct intense regions at ∼5.00 and 6.25 eV. Likewise, the shapes of the DOSs at both levels of theory reproduce similar peaks and shoulders, though with slight shifts in the relative energies. Perhaps the most noticeable discrepancies are the slight overestimation of the energy of the dark states of thymine, corresponding to the first band

in the DOS (4.2 eV with ADC(2) and 4.4 eV with CAM-B3LYP) and the minimum in the ADC(2) DOS at 5.5 eV, where the pure CT states appear on the CAM-B3LYP spectrum and DOS. The corresponding CT states at the former level of theory appear at higher energies (∼5.8 eV) and are mixed with local excitations of thymine, contributing to the band at 5.8 eV, rather than showing a steadier decay as in the CAM-B3LYP DOS. At any rate, we consider the observed energy shifts (∼0.2 eV) can be acceptable within TD-DFT, and do not alter significantly the conclusions derived in this work.

3 QM/MM configuration sampling absorption spectra

The absorption spectra of DNA and the DNA-PABA complex were obtained by computing the 30 first vertical absorption energies and oscillator strengths on top of an ensemble of equispaced frames from the corresponding 100 ns MD simulations. The QM region in these spectra were the 4 central nucleobases (not the nucleoside) and the 4 central nucleobases and PABA, respectively. The rest of the system (namely, sugars and phosphates, the rest of nucleotides and the water environment) was modelled at the molecular mechanics level via electrostatic embedding QM/MM. For comparison purposes, we also computed the configuration sampling spectra of PABA in water and intercalated in the strand, where only PABA was treated at the QM level. The spectra in each of the ensembles were calculated using Amber[12], for the MM part, interfaced with Orca 4.2,[8] for the QM part. The number of 100 configurations in the ensemble was selected based on the convergence of the averaged energy weighted by the oscillator strengths, shown in Figure S3. The resulting absorption spectra (Figure 2) are the convolution of Gaussian functions (Equation 1), each one centred on each absorption line (ΔE_{ci}), with a width (δ) of 0.085 eV and intensities proportional to the oscillator strengths (f_{ci}). The maximum absorption intensity of the resulting spectrum is scaled to 1.

$$Abs(E) = \sum_{c}^{N_{conf}} \sum_{i}^{N_{states}} f_{ci} e^{\frac{-(E-\Delta E_{ci})^2}{\delta^2/2}} \quad (1)$$

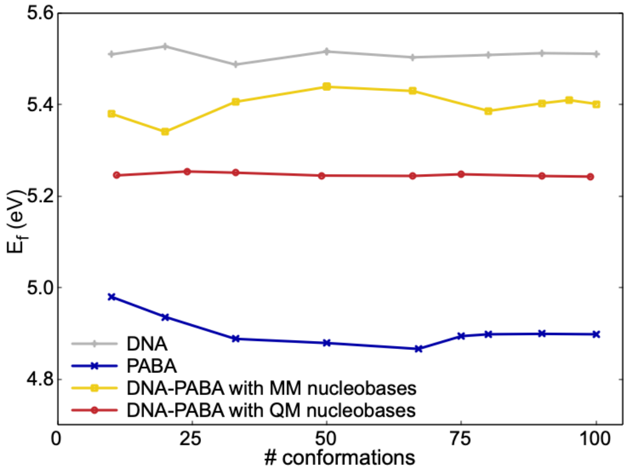

Figure S3: Convergence of the average energy of the spectra with respect to the number of snapshots from the MD simulation used to obtain the spectra.

The nuclear ensemble approach used in this work yields reasonable results for the absorption spectrum of PABA in water solution (Figure S4), as evidenced in the com-

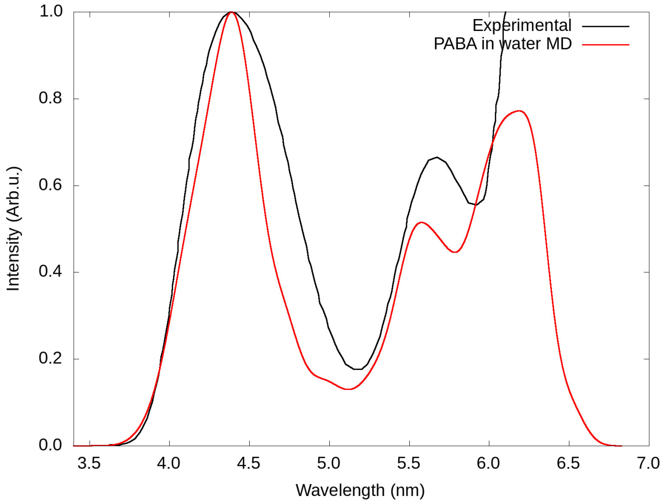

Figure S4: Experimental[13] and computed absorption spectra of unbound PABA in water solution. Theoretical spectrum computed from 100 vertical spectra at the QM(CAM-B3LYP/aug-cc-pVDZ)/MM level of theory and redshifted by 0.5 eV for a better coincidence with the experimental.

parison to the experimental spectrum, recorded in water at pH 4 by Chan et al.[13] The theoretical spectrum was obtained from the vertical absorption energies of 100 snapshots from a classical MD simulation of PABA surrounded by water molecules, analogously to the DNA and PABA-DNA spectra. We have thus estimated that for a larger QM system (4 nucleobases and one PABA molecule) the inaccuracies in the shape of the spectra associated to classical thermal sampling[14] are negligible in the context of this research. The lowest-energy band of the absorption spectrum of PABA intercalated in the DNA strand is redshifted with respect to that in water. This stabilisation of the excited states can be explained by either specific interactions with the nucleobases or by geometrical restrictions imposed by the stacking interactions. To assess the effect of the latter we removed the DNA from the intercalated PABA MD simulation and added water to model at the electrostatic embedding Molecular Mechanics level the solvent differences. The resulting spectrum is shown in grey in Figure S5. Since this spectrum is comparable to that of unrestrained PABA in water, we conclude that the redshift observed in the DNA is mainly due to interactions with the nucleobases. This is consistent with the farther redshift observed when including the flanking nucleobases in the QM region.

4 Analysis of the electronically excited states

In general terms, the characterisation of the excited states was based on the descriptors provided by the TheoDORE code, as explained in the main text and summed up in Scheme 1. We refer to the original paper [15] for mathematical grounds of these descriptors. The main limitation encountered for these descriptors was the isolation of the main contributions to the excitations when they are highly delocalised, as in excitons or excimers. In these cases, and considering the amount of excited states in hand, we deemed more convenient to study the electron-hole correlation matrices (Ω) directly. Each Ω_{AB} element indicates the probability of the transition of one electron from fragment A and to fragment B, as indicated in Eq. 2

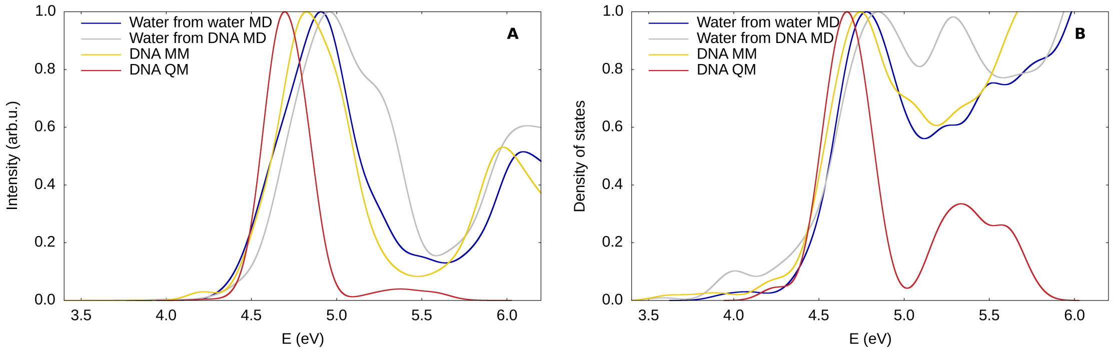

Figure S5: (A) Absorption spectra and (B) Density of States of PABA in explicit water solvation from a free movement MD (blue) and from the geometries of the intercalated MD (grey) and in the DNA-PABA complex with (red) and without (yellow) the flanking nucleobases included in the QM region. Computed from 100 vertical spectra at the QM(CAM-B3LYP/aug-cc-pVDZ)/MM level of theory.

$$\Omega_{AB} = \int_A \int_B |\gamma_{0I}(r_h, r_e)|^2 dr_h dr_e \tag{2}$$

where $\gamma_{0I}(r_h, r_e)$ is the 1-electron transition density matrix between electronic states 0 and I, r_h is the position of the hole, restricted to fragment A and r_e is the position of the electron restricted to fragment B. The integrals in Eq. 2 were computed as in Löwdin state analysis. In the resulting matrix, excitations with local character have larger diagonal elements (Ω_{AA}) whereas charge transfer excitations have larger off-diagonal elements ($\Omega_{AB,A\neq B}$). By locating the elements with larger contributions we characterised the excitations within excitons and charge-separated states.

4.1 Charge-separated states.

As occurs with local states, the positions of the electron and the hole in pure CT states can be easily identified with the position descriptors of the TheoDORE code. In contrast, the classification of the excitations in excimers is more complicated owing to their high delocalisation. For this reason, instead of classifying the states individually, as we did with excitons, we analysed the trends within each excitation type by generating cumulative electron-correlation matrices (Figure S6), whereby we get a qualitative identification of the dominant fragments involved in the excitations. We calculated these plots by summing the Ω_{AB} elements of all the excitations, previously classified in CT states or excimers using the criteria in Scheme 1.

The higher coefficients in the cumulative electron-hole correlation plots for A→T electron transfer reveal that these transitions dominate over the rest of pure DNA-DNA CT transitions, both in free DNA and the DNA-PABA complex. Likewise, PABA→T transitions are more relevant than PABA→A. Additionally, we observe that excimers have mainly a local character, with different CT contributions, mostly involving A→T and PABA→T transitions.

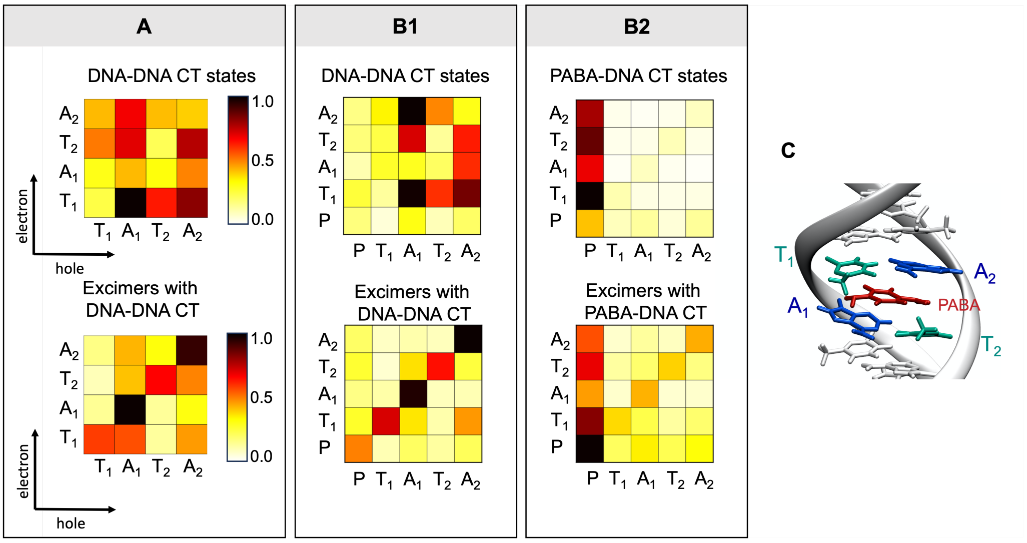

Figure S6: Cumulative electron-hole correlation plots of pure CT states and excimers in (**A**) the DNA strand and the DNA-PABA complex, considering (**B1**) the native states of DNA and (**B2**) states involving PABA. The color scale is relative to the maximum accumulated coefficient. All plots have the same axes and colour scale, only shown in panel A, for simplicity. (**C**) Section of the DNA-PABA complex, showing the fragments included in the calculation. The same numbering of the nucleobases is used in both the DNA duplex and the DNA-PABA complex.

4.2 Excitons.

In the case of excitons, as the main contributions to the excitation lie on the diagonal of the Ω matrix, only those elements were considered. The average delocalisation of the exciton determines the number of relevant Ω_{AA} elements. The average delocalisation may be biased by small contributions (e.g. D_{av}=2 might indicate both that two fragments contribute similarly to the excitation or that there is one heavier fragment and smaller contributions from the remaining ones), thus we calculated the number of high-contributing fragments as well. We considered these elements to be sufficiently relevant if their value was at least 50% of that of the highest Ω_{AA} element. In this way, we classified the DNA and DNA-PABA excitons according to the number of high-contributing locally-excited fragments (1-4 and 1-5, respectively) and, we identified them by locating the highest contributions (A, T or PABA).

From Table S2, one can see that from all the excitons in the DNA strand, around 80% are centred in 1-nucleobase, 18% are delocalised over 2 bases and 2% over 3 bases. None reach a significant simultaneous population of the four nucleobases in our sampling. These percentages shift to 87:12:1% in the DNA-PABA complex. Likewise, PABA only participates in 22% of the DNA-PABA excitons, meaning that the majority of excitons (78%) are alike those in the DNA strand. Using the classification in Table S2, we can estimate the participation of the different types of excitons in the absorption band.

5 CT assessment through electronic energies

As mentioned in the article, the likelyhood of formation of the radical anions and radical cations in charge transfer processes was estimated from the electronic energies of all the

Table S2: Classification of excitons according to the highest-contributing locally-excited fragments. The combinations not showed have no contribution. The nucleobases are numbered according to Figure S6C.

Number of fragments	T_1	A_1	T_2	A_2	Abundance (%)	T_1	A_1	T_2	A_2	PABA	Abundance (%)
1	X	-	-	-	19.87	X	-	-	-	-	15.84
	-	X	-	-	20.68	-	X	-	-	-	20.44
	-	-	X	-	15.95	-	-	X	-	-	17.04
	-	-	-	X	24.32	-	-	-	X	-	17.38
						-	-	-	-	X	16.52
2	X	-	X	-	1.22	X	-	X	-	-	0.17
	-	X	-	X	5.00	-	X	-	X	-	0.51
	-	X	X	-	2.43	-	X	X	-	-	2.73
	X	-	-	X	2.84	X	-	-	X	-	2.73
	-	-	X	X	2.84	-	-	X	X	-	0.85
	X	X	-	-	3.24	X	X	-	-	-	0.17
						X	-	-	-	X	1.36
						-	X	-	-	X	0.68
						-	-	X	-	X	1.19
						-	-	-	X	X	1.70
3	X	X	X	-	0.41						
	X	X	-	X	0.27						
	X	-	X	X	0.27						
	-	X	X	X	0.68						
						-	X	X	-	X	0.34
						X	-	X	-	X	0.34

Table S3: Vertical ionization energies (VIE), electron affinities (VEA), attachment energies (VAE) and detachment energies (VDE) in eV of PABA, adenine and thymine computed at the UKS-CAM-B3LYP/aug-cc-pVDZ level of theory in CPCM water.

	PABA	Thymine	Adenine
VIE	5.90	6.71	6.31
VEA	-1.56	-1.55	-1.15
VAE	-5.54	-6.14	-5.84
VDE	2.22	2.39	1.77

species, computed considering CPCM water solvation at the UKS-CAM-B3LYP/aug-cc-pVDZ level of theory as implemented in Orca 5.0.1. Vertical ionization energies (VIE) indicate the energy balance of the formation of the radical cations from the neutral species in their equilibrium geometries. Likewise, vertical electron affinities (VEA) correspond to the formation energies of the radical anions. Moreover, to evaluate the stability of these radical species, we computed the vertical attachment (VAE) and detachment energies (VDE), this is, the energy required to recover the neutral species from the equilibrium geometries of the cation and anion radical species, respectively.

Combining these data we can estimate the energy balance of an A→ B electron transfer (E_{CT}), calculated according to Eq. 4. Likewise, we estimated the energies released in the mutual quenching of the radicals (E_Q), calculated using Eq. 5. E_{CT} and E_Q for all the possible CT combinations in the DNA-PABA complex are shown on the left and right panels of Table S4, respectively.

Table S4: Charge transfer energies (E_{CT}), in eV, for all possible electron transfers.

	E_{CT} (eV)			E_Q (eV)		
	PABA$^{\cdot+}$	T$^{\cdot+}$	A$^{\cdot+}$	PABA$^{\cdot+}$	T$^{\cdot+}$	A$^{\cdot+}$
PABA$^{\cdot-}$	–	5.16	4.75	–	-3.92	-3.62
T$^{\cdot-}$	4.36	5.17	4.77	-3.15	-3.75	-3.46
A$^{\cdot-}$	4.76	5.57	5.17	-3.76	-4.37	-4.07

$$A + B \rightarrow A^{\cdot+} + B^{\cdot-} \tag{3}$$

$$E_{CT(A\rightarrow B)} = VIE_A + VEA_B \tag{4}$$

$$E_{Q(A\rightarrow B)} = VAE_{A^{\cdot+}} + VDE_{B^{\cdot-}} \tag{5}$$

From these data we conclude that PABA→T, yielding $PABA^{\cdot+}$ and $T^{\cdot-}$ is the most favourable CT process, as it has the lowest E_{CT}, 4.36 eV. Furthermore, the $PABA^{\cdot+}$ and $T^{\cdot-}$ radicals have the highest E_Q, -3.15 eV. Since their extinction releases less energy than any other radical combination, we expect them to be the longest lived pair of radicals.

References

[1] A. D. Becke, "Density-functional thermochemistry. iii. the role of exact exchange," *The Journal of Chemical Physics*, vol. 98, pp. 5648–5652, 7 Apr. 1993, ISSN: 0021-9606. DOI: 10.1063/1.464913.

[2] T. Yanai, D. P. Tew, and N. C. Handy, "A new hybrid exchange–correlation functional using the coulomb-attenuating method (cam-b3lyp)," *Chemical Physics Letters*, vol. 393, pp. 51–57, 1-3 Jul. 2004, ISSN: 00092614. DOI: 10.1016/j.cplett.2004.06.011.

[3] Y. Zhao and D. G. Truhlar, "The m06 suite of density functionals for main group thermochemistry, thermochemical kinetics, noncovalent interactions, excited states, and transition elements: Two new functionals and systematic testing of four m06-class functionals and 12 other functionals," *Theoretical Chemistry Accounts*, vol. 120, pp. 215–241, 1-3 2008.

[4] J.-D. Chai and M. Head-Gordon, "Systematic optimization of long-range corrected hybrid density functionals," *The Journal of Chemical Physics*, vol. 128, 8 Feb. 2008, ISSN: 0021-9606. DOI: 10.1063/1.2834918.

[5] J.-D. Chai and M. Head-Gordon, "Long-range corrected hybrid density functionals with damped atom–atom dispersion corrections," *Physical Chemistry Chemical Physics*, vol. 10, pp. 6615–6620, 44 2008. DOI: https://doi.org/10.1039/B810189B.

[6] T. H. Dunning, "Gaussian basis sets for use in correlated molecular calculations. i. the atoms boron through neon and hydrogen," *The Journal of Chemical Physics*, vol. 90, pp. 1007–1023, 2 Jan. 1989, ISSN: 0021-9606. DOI: 10.1063/1.456153.

[7] V. Barone and M. Cossi, "Quantum calculation of molecular energies and energy gradients in solution by a conductor solvent model," *The Journal of Physical Chemistry A*, vol. 102, pp. 1995–2001, 11 Mar. 1998, ISSN: 1089-5639. DOI: 10.1021/jp9716997.

[8] F. Neese, F. Wennmohs, U. Becker, and C. Riplinger, "The orca quantum chemistry program package," *The Journal of Chemical Physics*, vol. 152, p. 224 108, 22 Jun. 2020, ISSN: 0021-9606. DOI: 10.1063/5.0004608.

[9] F. Neese, "Software update: The orca program system—version 6.0," *WIREs Computational Molecular Science*, vol. 15, 2 Mar. 2025, ISSN: 1759-0876. DOI: 10.1002/wcms.70019.

[10] F. Weigend, "Accurate coulomb-fitting basis sets for h to rn," *Physical Chemistry Chemical Physics*, vol. 8, p. 1057, 9 2006, ISSN: 1463-9076. DOI: 10.1039/b515623h.

[11] G. L. Stoychev, A. A. Auer, and F. Neese, "Automatic generation of auxiliary basis sets," *Journal of Chemical Theory and Computation*, vol. 13, pp. 554–562, 2 Feb. 2017, ISSN: 1549-9618. DOI: 10.1021/acs.jctc.6b01041.

[12] D. A. Case, K. Belfon, I. Y. Ben-Shalom, *et al.*, *Amber 2020*, University of California, San Francisco, 2020.

[13] C. T. L. Chan, C. Ma, R. C. T. Chan, *et al.*, "A long lasting sunscreen controversy of 4-aminobenzoic acid and 4-dimethylaminobenzaldehyde derivatives resolved by ultrafast spectroscopy combined with density functional theoretical study," *Physical Chemistry Chemical Physics*, vol. 22, pp. 8006–8020, 15 Apr. 2020, ISSN: 14639076. DOI: 10.1039/c9cp07014a.

[14] M. Barbatti and K. Sen, "Effects of different initial condition samplings on photodynamics and spectrum of pyrrole," *International Journal of Quantum Chemistry*, vol. 116, pp. 762–771, 10 May 2016, ISSN: 0020-7608. DOI: 10.1002/qua.25049.

[15] F. Plasser, “Theodore: A toolbox for a detailed and automated analysis of electronic excited state computations,” *The Journal of Chemical Physics*, vol. 152, p. 084 108, 8 Feb. 2020, ISSN: 0021-9606. DOI: 10.1063/1.5143076.
